# Long-range spontaneous droplet self-propulsion on wettability gradient surfaces

**DOI:** 10.1038/s41598-017-07867-5

**Published:** 2017-08-08

**Authors:** Chaoran Liu, Jing Sun, Jing Li, Chenghao Xiang, Lufeng Che, Zuankai Wang, Xiaofeng Zhou

**Affiliations:** 1 Science and Technology on Microsystem Laboratory, Shanghai Institute of Microsystem and Information Technology, Chinese Academy of Sciences, Shanghai, 200050 China; 2 Department of Mechanical and Biomedical Engineering, City University of Hong Kong, Hong Kong, 999077 China; 3 College of Biomedical Engineering and Instrument Science, Zhejiang University, Hangzhou, 310027 China; 4 School of Electronic, Electrical and Communication Engineering, University of Chinese Academy of Sciences, Beijing, 100049 China

## Abstract

The directional and long-range droplet transportation is of great importance in microfluidic systems. However, it usually requires external energy input. Here we designed a wettability gradient surface that can drive droplet motion by structural topography. The surface has a wettability gradient range of over 150° from superhydrophobic to hydrophilic, which was achieved by etching silicon nanopillars and adjusting the area of hydrophilic silicon dioxide plane. We conducted force analysis to further reveal the mechanism for droplet self-propulsion, and found that the nanostructures are critical to providing a large driving force and small resistance force. Theoretical calculation has been used to analyze the maximal self-propulsion displacement on different gradient surfaces with different volumes of droplets. On this basis, we designed several surfaces with arbitrary paths, which achieved directional and long-range transportation of droplet. These results clarify a driving mechanism for droplet self-propulsion on wettability gradient surfaces, and open up new opportunities for long-range and directional droplet transportation in microfluidic system.

## Introduction

The spontaneous and directional liquid droplet transportation on a solid surface without any externally applied force has attracted increasing interests in microfluidic systems, especially for analytical chemistry and bioassay applications in recent years^1–11^. Directed self-propulsion of droplets normally requires an energy supply (passive or active) to overcome the inherent contact line pinning or defects as well as the spatial asymmetry to rectify and sustain the transport of droplet in the preferential direction^12, 13^.

Elegant approaches to break the wettability symmetry of a droplet on a surface have been developed by leveraging on the gradients of chemical^2, 7, 14, 15^, structural topography^16–21^, temperature^22^, electric force^23–26^, mechanical vibration^12, 27^, PH-induced^28, 29^ or their combinations^30–33^. Among these strategies, the creation of wettability gradient by structural topography or chemical heterogeneity has gained increasing attention owing to its advantages such as the alleviation of external energy supply and easy operation^13, 28, 34–41^. In the aspect of chemical heterogeneity, the spontaneous droplet motion resulting from the flat surface with a wettability gradient is first experimentally demonstrated by Chaudhury and Whitesides^1^. By allowing the vapor of decyltrichlorosilane to diffuse over a silicon wafer, the droplet can move uphill. The grading of the chemical functional group density was recently extended to 2-D materials such as graphene, which can drive the droplet to move a total of 1–3 mm in one direction^25^. The motion of liquid droplets on chemically defined radial wettability gradients consisting of alternating wettability, i.e., hydrophilic and hydrophobic was also reported^42, 43^. Despite extensive progress, chemical gradient surfaces involve the functionalization of the surface with molecular gradient of alkanethiolate or alkylsilane, which may deteriorate due to migration or degradation of organic molecules and result in decay of chemical gradient in the long term operation. Thus, the directional droplet transport purely driven by the structural topography provides a compelling strategy to address this problem.

The long-range transport demands a large wetting contact angle (CA) gradient and a small contact angle hysteresis. To reduce the contact angle hysteresis and enlarge the wettability gradient range, a superhydrophobic surface is preferred which normally requires a hydrophobic coating^44^. While the hydrophobic coating provides a barrier for fabricating wettability gradient ranges from hydrophilicity to superhydrophobicity. As a result, the wettability gradient range based on the hydrophobic coating ranges from 100° to 150°^43^, which dramatically limits the droplet transport distance. Thus, to enlarge the wettability gradient, an intrinsically superhydrophilic surface is desired, however, the viscous energy dissipation imposed on such a design is large. Wang^45^ and Checco^46^ experimentally demonstrated the tapered cone nanotextures surfaces exhibits the largest macroscopic contact angles and the weakest hysteresis, which can reduces the viscous energy dissipation^47^. To decouple the conflicting requirement, in this work we adopt this nanotextures surfaces and etch a 3D nanoscale silicon pillars structure as the superhydrophobic region. And the plane SiO_2_ is selected as the hydrophilic region which achieves a larger wettability gradient ranging from 15.5° to 166.0 100 100°. The designed wettability gradient path surfaces provide a discrete reduced CA by increasing the area fraction of plane SiO_2_ which can impart a wettability gradient range over 150° for a continuous and spontaneous droplet motion. In general, surfaces exhibit contact angle hysteresis that provides an additional energy barrier for droplet motion. The nanoscale silicon pillars structure has a hysteresis CA 3.1°, which optimizes the pinning of droplet advancing and receding lines. We further analyze the maximal self-propulsion displacement on different gradient surfaces with different volumes of droplets by theoretical calculation which is consistent with our experimental results. And more droplet motion paths besides the reported path are achieved by this surface wettability gradient method.

## Results and Discussion

**Figure 1:**
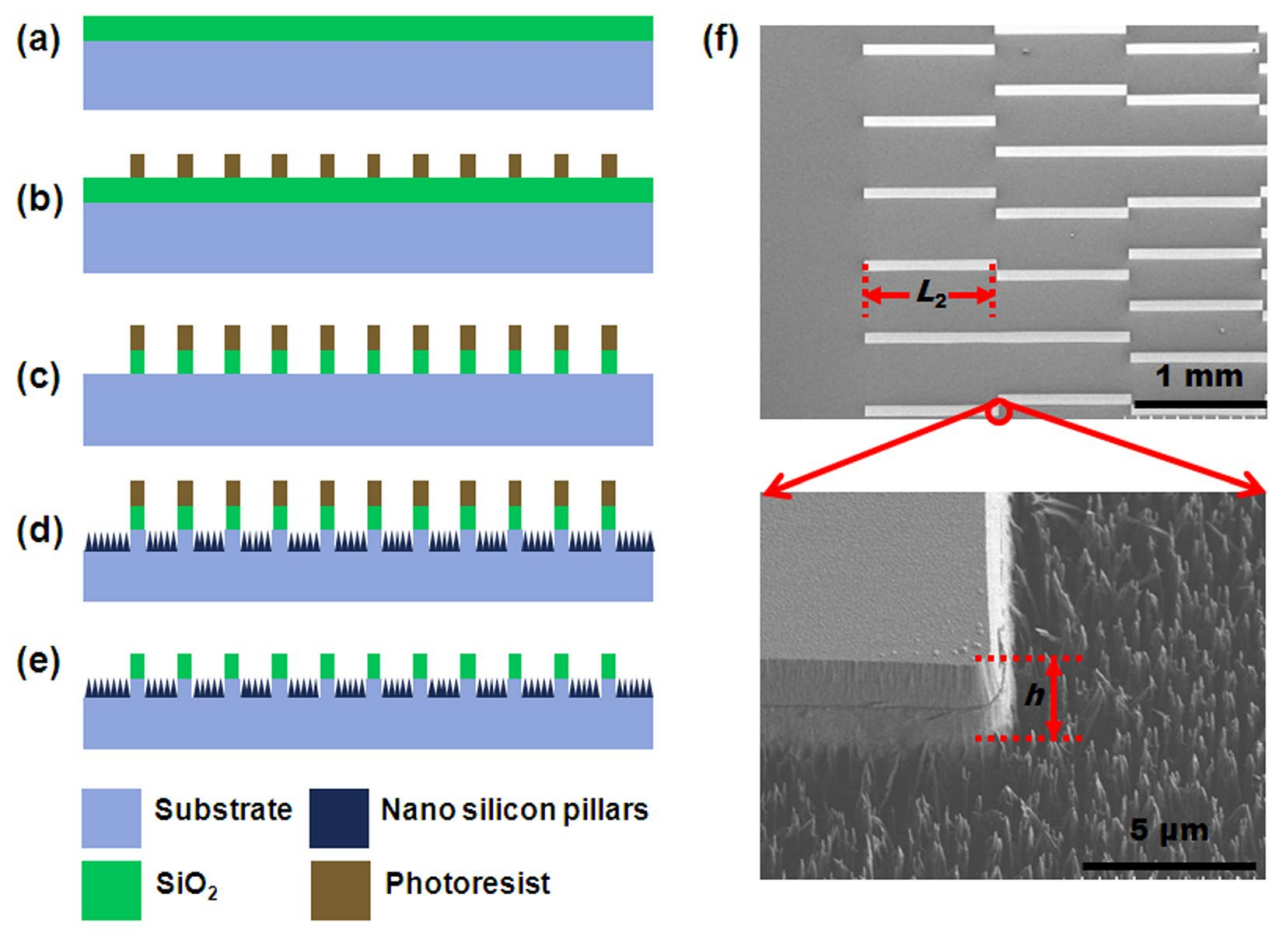
Fabrication process of wettability gradient surface. (**a**) Oxidation. (**b**) Patterns fabrication though UV lithography. (**c**) Silicon oxide etching via RIE. (**d**) Silicon etching via deep RIE. (**e**) Removal of photoresist. (**f**) SEM images of the patterned wettability gradient surface, SiO_2_ stripe and the silicon nanopillars structures.

**Figure 2:**
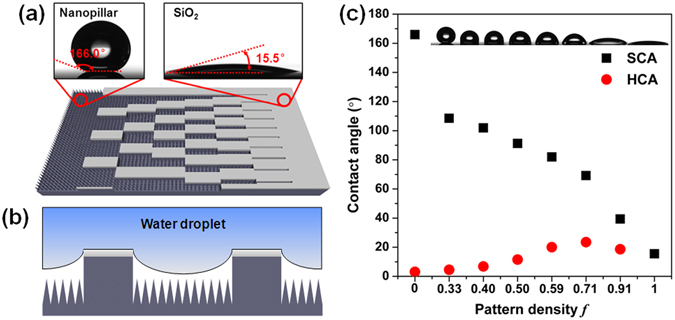
Surface design and wettability characterization. The size of all the SiO_2_ stripe is 1 mm × 90 μm × 2 μm. (**a**) Top view schematic of the gradient surface. From left to right, the pattern densities are 0, 0.33, 0.40, 0.50, 0.59, 0.71, 0.91, 1, and the space between SiO_2_ stripes are 180 μm, 135 μm, 90 μm, 63 μm, 36 μm, 9 μm, 0 μm, respectively. (**b**) Undercut structure of solid-liquid contact region. (**c**) The static contact angle (SCA) and hysteresis contact angle (HCA) of water droplet on each region of the gradient surfaces.

To achieve the spontaneous droplet motion, we designed patterned surface with wettability gradient (Figs 1f and 2a). The surface is composed of regions with uniform SiO_2_ stripes and silicon nanopillars. The top surface is made of plane SiO_2_ with water static CA of 15.5° (Fig. 1). The valleys of the stripes are covered silicon nanopillars fabricated by deep reactive ion etching (RIE), which exhibits a static CA of 166.0°. To quantify the relative hydrophobicity of the patterns, we introduced a pattern density *f* as Fig. 2b shows.

1 $$f=\frac{{A}_{{{\rm{S}}{\rm{i}}{\rm{O}}}_{2}}}{{A}_{{{\rm{S}}{\rm{i}}{\rm{O}}}_{2}}+{A}_{{\rm{S}}{\rm{N}}{\rm{P}}}}$$

here, *A*_SiO2_ and *A*_SNP_ are the area of SiO_2_ region (hydrophilic) and silicon nanopillars region (superhydrophobic), respectively. Thus the pattern density of the surface gradually increases from left to right, which leads to a wettability gradient ranging from superhydrophobic to hydrophilic regions. And the measured static CAs are consistent with the ones calculated by Cassie-Baxter model^46^ (see Table S1).

Figure 1f shows the SEM image of the as-fabricated surface. The size of each SiO_2_ stripe is 1 mm length × 90 μm width × 2 μm height. And the spacing between SiO_2_ stripes decreases from 180 μm to 0 μm along wettability gradient direction, thus the pattern density of the surface increases from 0 to 1, accordingly. We also designed several surfaces with different wettability gradient ranges and stripe lengths.

We measured the static CA and hysteresis CA of a droplet on each region using OCA15EC Drop Shape Analysis (Dataphysics, Germany). Figure 2c shows the static contact angle and hysteresis contact angle profiles along the wettability gradient direction. It can be seen that the static CA reached up to 166.0° at superhydrophobic side and decreased gradually to 15.5° at hydrophilic side as the pattern density increases from 0 to 1. The top image shows that the real shape of droplets along the gradient direction, the shape of droplets changed from spherical segment to thin film while the static CA changed from 166.0° to 15.5° accordingly. Unlike other wettability gradient surfaces with a range less than 120°^37, 43, 48^, the superhydrophobic and hydrophilic region on two ends greatly enlarged the wetting CA gradient range over 150°.

**Figure 3:**
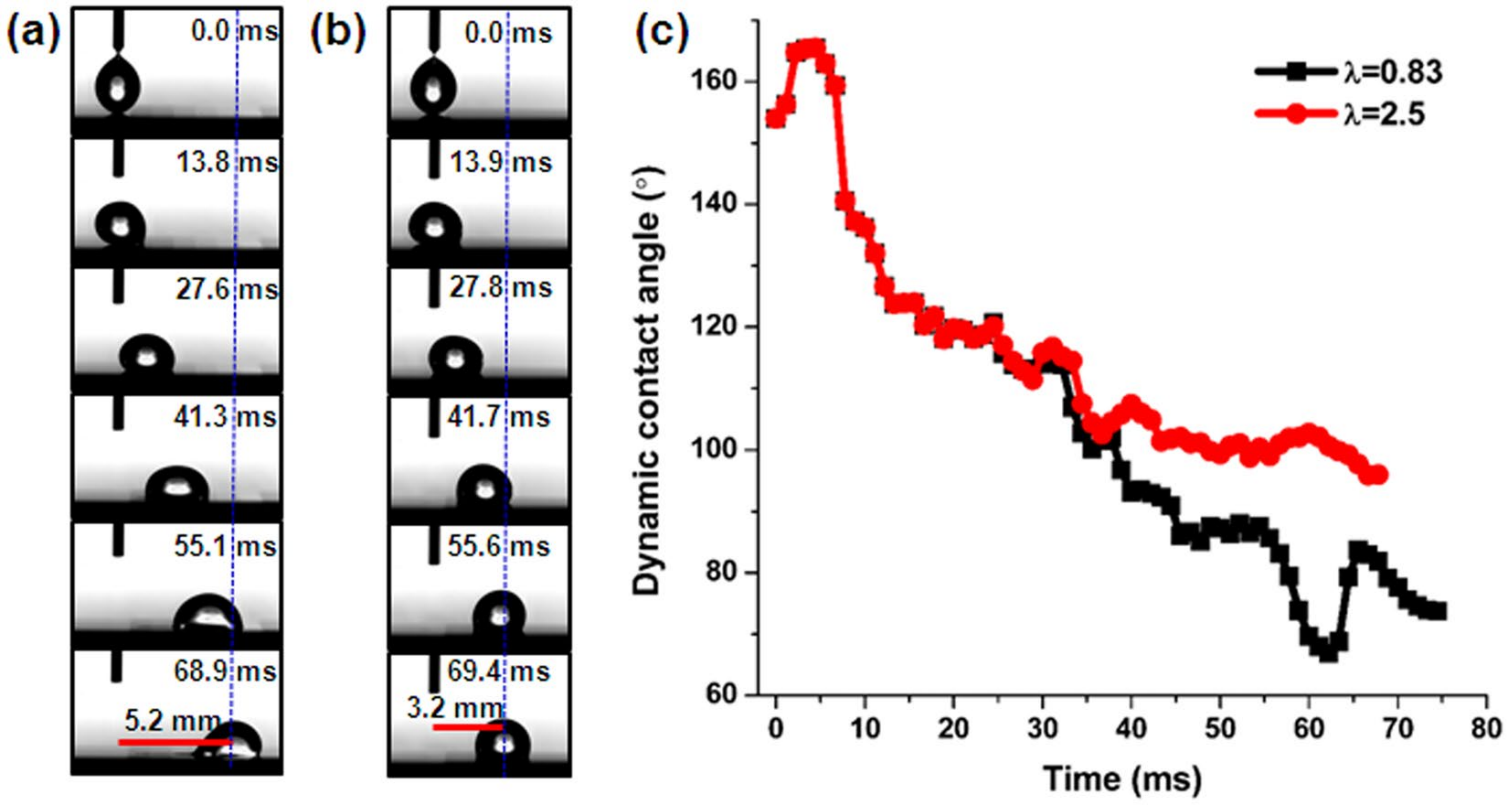
Time-resolved images of a 7 μL water droplets spontaneously moving along surfaces with different λ (ratio of stripe length and droplet radius). (**a**) λ = 0.83. (**b**) λ = 2.5. (**c**) The variation of dynamic contact angle as a function of time.

To examine the effect of the as-prepared CA gradient surface on droplet motion, we conducted two contrast experiments, and introduced a dimensionless parameter *λ* as the ratio of stripe length *L*_2_ and droplet radius *r*, i.e., *λ* = *L*_2_/*r*. Figure 3a and b show a series of dynamic images of a 7 μL water droplet gently placed on the border of the first two regions. The droplet moved from static state and rapidly accelerated toward the more wetting direction of the gradient surface, and finally attained a stable state (see from videos ‘*λ* = 0.83 wettability gradient surface’ and ‘*λ* = 2.5 wettability gradient surface’). The droplet velocity in self-propulsion has been measured and the results can be found in Fig. S5. The droplet velocity is nonlinear due to the discrete wettability gradient surface. For *λ* = 0.83, the droplet moved at a mean velocity of 75 mm/s and achieved a maximum displacement of 5.2 mm. And for *λ* = 2.5, the droplet moved at a mean velocity of 46 mm/s and only reached a maximum displacement of 3.2 mm. With the time progression, the dynamic contact angle *θ*_d_ also decreased from 165.5° to 66.9° and 165.5° to 96.0° for *λ* = 0.83 and 2.5, respectively.

To further explore the underlying mechanism for droplet self-propulsion, we conducted force analysis. As commonly observed, the shape of a droplet depends on its size. Small droplets make spherical caps, while larger ones would be flattened by the effect of gravity if the droplet size larger than capillary length ${\beta }^{-1}=\sqrt{{\gamma }_{{\rm{L}}{\rm{V}}}/\rho g}$ (2.73 mm for water). The spherical surface of the droplet is due to the surface tension dominating gravity, which tends to impose a minimal surface^49, 50^. And a 7 μL droplet (diameter 2.37 mm < *β* ^−1^) is selected to ignore the gravity in our experiments and force analysis of the droplet motion. Equation 2 results from an analysis of force of a droplet moving on a patterned surface. The driving force (*F*_d_) for the droplet movement on a heterogeneous surface results from the variation in the wettability property (or surface energy) of the liquid-solid interface, while the resistance force comes from two sources: one is the hysteresis force, *F*_h_, originating from the hysteresis phenomenon of droplet motion, and the other is the viscous resistance force, *F*_v_, during the droplet motion^2^

2 $${\rm{\Sigma }}F={F}_{{\rm{d}}}-{F}_{{\rm{h}}}-{F}_{{\rm{v}}}=ma$$

The driving force a water droplet on the gradient surface can be expressed as^51^

3 $${F}_{{\rm{d}}}\cong {\gamma }_{{\rm{L}}{\rm{V}}}{R}_{{\rm{b}}}^{2}\pi \frac{{\rm{d}}\,\cos \,{\theta }_{{\rm{d}}}}{{\rm{d}}x}$$

where *γ*_LV_ represents the surface tension of the droplet, *R*_b_ is the base radius of the droplet in contact with the solid, *θ*_d_ is the dynamic contact angle of the water droplet on solid surfaces. We assume the contact area as nearly a circle to calculate (see from Supplementary Information). This equation reveals that the driving force is proportional to contact angle gradient d cos *θ*_d_/d*x* and solid-liquid contact area *R*_b_^2^. To activate motion, the droplet should overcome a moving barrier due to contact angle hysteresis, the hysteresis force is expressed as^51^

4 $${F}_{{\rm{h}}}=2{\gamma }_{{\rm{L}}{\rm{V}}}{R}_{{\rm{b}}}(\cos \,{\theta }_{{\rm{o}}{\rm{r}}}-\,\cos \,{\theta }_{{\rm{o}}{\rm{a}}})$$

here, *θ*_oa_ and *θ*_or_ are the advancing and receding CA at the center of the droplet, respectively. When the droplet moves on the patterned surface, the viscous resistance force generated within the liquid can be expressed as^49^

5 $${F}_{{\rm{v}}}={\int }_{{\rm{A}}}^{{\rm{B}}}{\sigma }_{xz}(0)f{r}_{w}{\rm{d}}x=1.5\pi {R}_{{\rm{b}}}{\gamma }_{{\rm{L}}{\rm{V}}}\eta V{\int }_{{\rm{A}}}^{{\rm{B}}}\frac{f{r}_{w}}{{h}_{x}}{\rm{d}}x$$

where *σ*_*xz*_(0) is the viscous stress at solid/liquid interface, *r*_*w*_ is the roughness of the SiO_2_ plane, *h*_*x*_ is the height of droplet along the motion direction, *η* is the viscosity of the liquid and *V* is the velocity of the moving droplet, *f* represents pattern density of each wettability regions. Points A and B are the two endpoints of droplet (see from Supplementary Information). According to equation 3~5, increasing CA gradient and reducing CA hysteresis could improve driving force and reduce resistance force, thus promote droplet motion.

**Figure 4:**
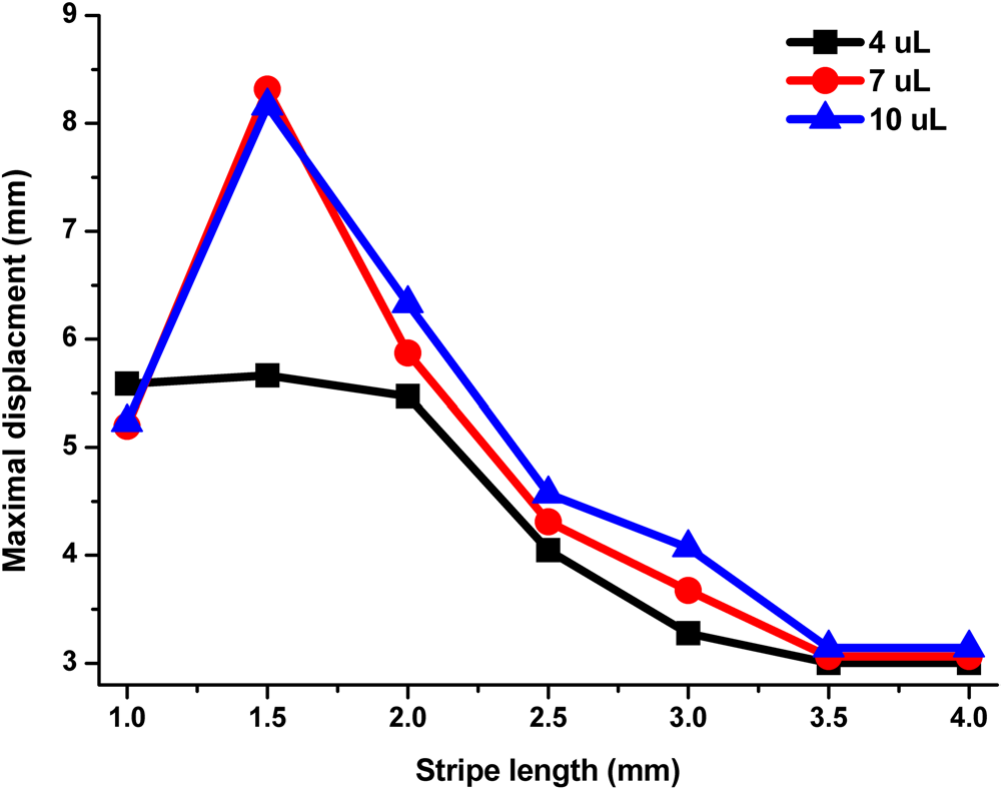
Theoretical calculation of the maximal motion displacement of different volume droplets self-propulsion on different stripe length *L*_2_ gradient surfaces with wettability range from 166° to 39.4°, and each pattern density in gradient is 0, 0.33, 0.40, 0.50, 0.59, 0.71 and 0.91, respectively.

During the droplet self-motion process, droplet kinetic energy depends on the accumulated works applied by the driving, hysteresis and viscous forces. To further understand the droplet self-motion, theoretical calculation is used to analyze the maximal droplet displacement with different stripe length *L*_2_ and droplet volumes (4 μL, 7 μL,10 μL) on the gradient surfaces with wettability range from 166.0° to 39.4°, and each pattern density in gradient is 0, 0.33, 0.40, 0.50, 0.59, 0.71 and 0.91, respectively. Despite the wetting profile of gradient surface is discrete, the forces acting on the dynamic droplets were simplified and defined points at middle positions of two (four) adjacent wettability gradient regions were used to calculate the driving and hysteresis forces (Fig. S2). And the work of these forces can be calculated by, $W=\sum F{\rm{\Delta }}x$ where ${\rm{\Sigma }}F$ is the resultant force at a defined position, and Δ*x* is the displacement between two adjacent positions. In the calculation, the additional energy resulting from the descending of droplet barycenter has been considered. And the droplet will arrive at the maximal displacement when the kinetic energy decreasing to zero. Figure 4 shows the calculation of the maximal displacement of three volumes droplets moving on the gradient surfaces with different stripe length. As the results shows, the droplets can move toward the end of the gradient surface when the stripe length *L*_2_ is 1 mm. However the droplet cannot move toward the end of the gradient surface with stripe length increases to 2 mm. When the stripe length is longer than 3.5 mm, all three volumes droplet cannot move spontaneously because the kinetic energy is consumed before the advancing line overlaps the next wettability gradient.

**Figure 5:**
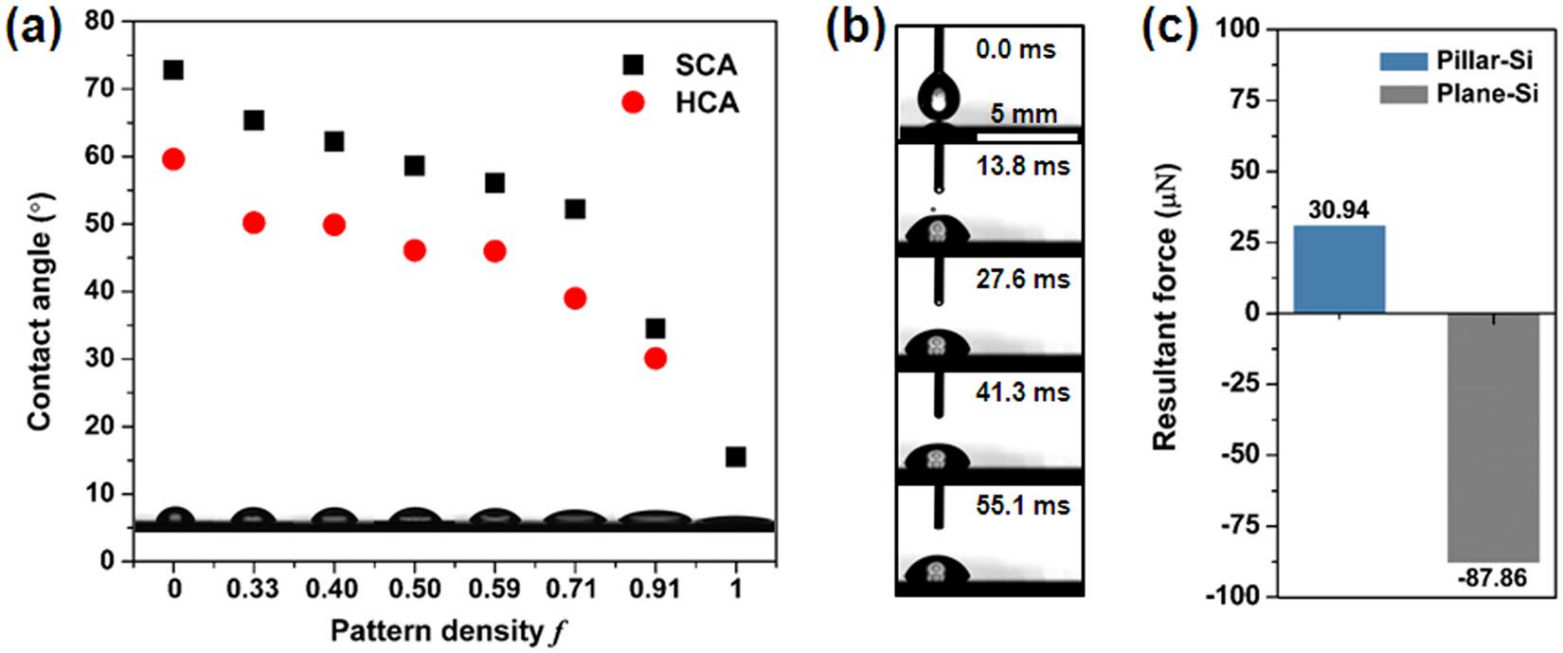
(**a**) The spatial variation of static contact angle (SCA) and the hysteresis contact angle (HCA) as a function of pattern density *f* for the surface without nanostructures. (**b**) Time-resolved images of a 7 μL water droplets moving on plane-Si gradient stripes with λ = 0.83. (**c**) The comparison of resultant force of droplet on two types of surfaces. Pillar-Si surface has nanostructures around SiO_2_ stripes. While on plane-Si surface, all the nanostructures are replaced by silicon plane.

We further elucidated the effect of nanostructure on the fast droplet transport. Given the ratio of stripe length and droplet radius λ as 0.83, we designed surfaces of two types, pillar-Si and plane-Si. The surfaces are composed of several regions with identical stripe length of 1 mm, and the pattern densities sequences are 0, 0.33, 0.40, 0.50, 0.59, 0.71, and 0.91. While pillar-Si surface has silicon nanopillars around the SiO_2_ stripes, as for plane-Si surface, all the silicon nanopillars were replaced by silicon plane. Plane-Si surface caused CA gradient range dramatically decreased to 57.34° in contrast of the pillar-Si surface (over 150°). Moreover, the first region on pillar-Si surface has a much smaller CA hysteresis (3.1°) than plane-Si surface (59.6°). To quantitatively analyze the effect of nanostructures on droplet motion, we calculated the driving force and hysteresis force when the droplet was placed on the boundary of the first two regions, and the resultant force (*F*_*d*_ − *F*_*h*_) were shown in Fig. 5c, the initial resultant force was 30.94 μN and −87.86 μN for pillar-Si and plane-Si surface, respectively. Thus the droplet on pillar-Si surface can overcome the energy barrier and move towards the higher wetting surface, and it should be noted that the negative force means there exists a much larger hysteresis resistance force that the droplet cannot move, which is consistent with our experimental results (see from video ‘λ = 0.83 wettability gradient surface without silicon nanopillars’). We observed that the droplet merely spread on the surface with a contact angle of 70°, and the droplet shape remained unchanged as time progresses. Taken together, these results demonstrate the importance of nanoscale structure to provide a large driving force and smaller resistance force.

**Figure 6:**
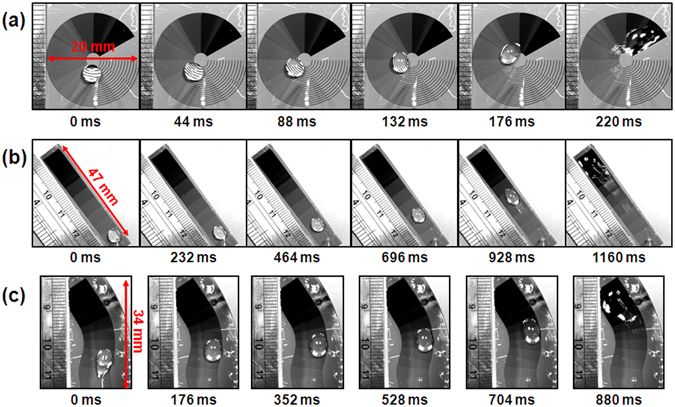
Three surfaces with arbitrary paths are designed for large volume droplet transport. The pattern density on these surfaces range from 0 to 1, and the CA gradient of the three surfaces are over 150°. (**a**) Droplet self-propulsion on annular path, which has an inner diameter of 3 mm and outer diameter of 20 mm, droplet could rotate about 210° around the center. (**b**) Droplet self-propulsion on straight path, the total length of the path is about 47 mm, and the droplet achieved a displacement of 37.5 mm (**c**) Droplet self-propulsion on ‘S’ shape path, the path has a length of 34 mm between two endpoints.

To further explore the directional and long-range droplet transport, we designed several wettability gradient surfaces with arbitrary paths, such as annular path, straight path, and ‘S’ path (see from videos ‘Annular path’, ‘Long straight path’ and ‘S path’). Figure 6 shows a series of dynamic images of a water droplet gently placed on the three wettability gradient surfaces with a static CA gradient more than 150°, as time progressed, the droplet shape continuously changed from sphere segment to thin film. The annular surface has an inner diameter of 3 mm and outer diameter of 20 mm, and it’s composed of twelve identical fan-shaped regions with varying wettability. The droplet has a large volume of 60 uL so that it can overlap several regions simultaneously. We observed that the droplet spontaneously rotated nearly 210° from hydrophobic side to hydrophilic side around the center (Fig. 6a). The straight path is composed of ten identical regions and it has a total length of 47 mm. The droplet spontaneously moved on the surface and achieved a maximum displacement of 37.5 mm (Fig. 6b). In addition, we designed paths with ‘S’ shape, which has a length of 34 mm between two endpoints. The droplet self-propelled and swerved on the ‘S’ path along gradient direction (Fig. 6c). The designed wettability gradient paths have a good reproducibility (see from Fig. S6 and video ‘Droplet flow’). These multi paths provide the capability to realize directional and long-range transportation of large volume droplets.

## Conclusions

In summary, we designed surfaces with wettability gradient by MEMS compatible process technology, which could achieve droplet self-propulsion without external force or energy applied. The varying wettability of the surface was achieved by modulating the area fraction of superhydrophobic silicon nanopillars and hydrophilic SiO_2_ stripes. We define the pattern density as the area fraction of SiO_2_ stripes on each region. The static contact angle decreases from 166.0° to 15.5° with the increasing of pattern density, and it achieved a large wettability gradient range of over 150°. We introduced a dimensionless parameter λ as the ratio of stripe length and droplet radius, and carried out experiments to investigate the motion behavior of droplet on the as-prepared surface. We found that droplet moved longer and faster with a smaller λ, the droplet moved with a mean velocity of 75 mm/s and 46 mm/s and achieved a displacement of 5.2 mm and 3.2 mm given λ = 0.83 and 2.5, respectively. To further explore the underlying mechanism for droplet self-propulsion, we conducted force analysis, and elucidated the effect of nanostructure on the fast droplet transport. By contrast, we prepared two types of surfaces with the same stripe length and pattern density. Pillar-Si surface has silicon nanopillars around SiO_2_ stripes, and the nanostructures were replaced by silicon plane on plane-Si surface. The experimental results showed that droplet on pillar-Si surface can overcome the energy barrier and move towards higher wetting surface, while the biggish hysteresis resistance force on plane-Si surface hindered the droplet motion. Therefore, the nanostructure on surface is critical in providing a large driving force and smaller resistance force for droplet. And theoretical calculation has been used to analyze the maximal self-propulsion displacement on different gradient surfaces with different volumes of droplets. On the basis of theoretical analysis and fabrication technology, we designed several surfaces to realize the directional and long-range droplet motion on arbitrary paths. These results reveal the underlying mechanism for droplet self-propulsion and present the potential for directional and long-range droplet transportation in microfluidic system, micropump needles, biochips, and so on.

## Methods

### Sample Fabrication

We employed standard Micro-Electro-Mechanical System (MEMS) process technology to fabricate roughness gradient structures on silicon surface, which consists of two essential structural and chemical features, silicon nanopillars, and silicon dioxide (SiO_2_) stripes. Figure 1 shows the fabrication procedure of the patterned surface. Briefly, to amplify the range of wettability gradient, we first fabricated a 2-μm-thick SiO_2_ film on the silicon wafer at high temperature (Fig. 1a). Then, we used photolithography process to selectively cover photoresist on SiO_2_ film (Fig. 1b), following by Reactive Ion Etching (RIE) to etch the SiO_2_ that are not protected by photoresist (Fig. 1c), and deep RIE was used to further etch the silicon substrate, thus formed silicon nanopillars (Fig. 1d). The deep RIE process included cyclic passivation and etching modes in which C_4_F_8_ and SF_6_ were used. In the etching cycle, the SF_6_ flow rate was ~130 sc cm and platen power was set at ~12 W. In the passivation cycle, the C_4_F_8_ flow rate was ~85 sc cm^52^. Finally, we removed the photoresist, the surface fabrication process was completed (Fig. 1e).

### Experiment Process

The liquid droplet was formed using a syringe pump (Lead Fluid TYD01-01) and gently released to the sample with the highest contact angle. A Photron FASTCAM SA4 high speed camera (Photron, Japanese) was employed to record the instantaneous movement of water droplet, and the frame rate was set as 3600 fps.

## Electronic supplementary material

Supplementary Information
