# Supplementary material for: Long-range spontaneous droplet self-propulsion on wettability gradient surfaces
Source: Sci Rep. 2017 Aug 8;7:7552. doi: 10.1038/s41598-017-07867-5 (PMC5548791; doi:10.1038/s41598-017-07867-5)
Supplement: Supplementary file 1 — Supplementary Information [file 41598_2017_7867_MOESM1_ESM.pdf]

Supplementary Information

Long-range spontaneous droplet self-propulsion on wettability gradient surfaces

Chaoran Liu[1, 4], *Jing Sun*[2], *Jing Li*[2], *Chenghao Xiang*[1, 4], *Lufeng Che*[3,1,*], *Zuankai Wang*[2, *], *Xiaofeng Zhou*[1,*]

1. Driving force and hysteresis resistance force

During the whole process, the difference of the Gibbs surface energy between its two sides acts as the driving force.

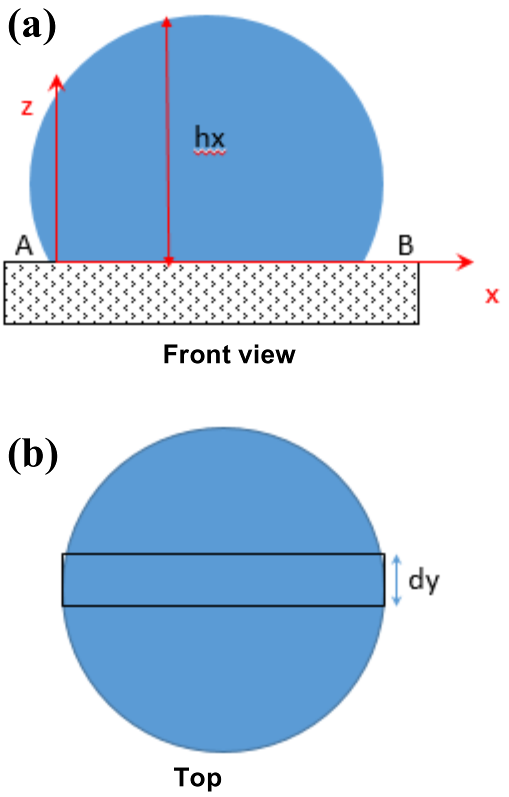

Figure S1. Schematic drawing shows a spherical segment water droplet on the wettability gradient stripes from the front view (a) and the top view (b).

As shown in the Fig. S1, if we take the ribbons with width dy into consideration, the driving force for the displacement dx can be expressed as[1]:

$$F_{d1} = -\frac{d\Delta G}{dx} = \gamma_{LV}\left(\cos\theta_{Ba} - \cos\theta_{Ar}\right) \quad \text{(S1)}$$

The overall driving force:

$$F_{d2} = \int \gamma_{LV}\left(\cos\theta_{Ba} - \cos\theta_{Ar}\right)dy \quad \text{(S2)}$$

Where θ_{Ar} and θ_{Ba} are the droplet receding CA of rear point A and advancing CA of front point B. It assumes that the water droplet on gradient stripes can be considered as a spherical segment and the contact area is a circle with radius R_b. Thus,

$$\begin{aligned} \cos\theta_{Ba} &= \cos\theta_{oa} + R_b \frac{d\cos\theta_d}{dx} \\ \cos\theta_{Ar} &= \cos\theta_{or} - R_b \frac{d\cos\theta_d}{dx} \end{aligned} \quad \text{(S3)}$$

With θ_{oa} and θ_{or} being the advancing and receding contact angle at the center of droplet, θ_d being the dynamic contact angle which can be calculated as: $\cos\theta_d = 1/2\left(\cos\theta_a + \cos\theta_r\right)$. Here θ_a and θ_r are the droplet advancing and receding CAs on a wettability gradient surfaces. Finally,

$$\begin{aligned} F_{d2} &= \int \gamma_{LV}\left(\cos\theta_{Ba} - \cos\theta_{Ar}\right)dy \\ &= \gamma_{LV}\pi R_b^2 \frac{d\cos\theta_d}{dx} + 2\gamma_{LV}R_b\left(\cos\theta_{oa} - \cos\theta_{or}\right) \\ &= F_d - F_h \end{aligned} \quad \text{(S4)}$$

where F_d and F_h are the driving force and hysteresis resistance force, γ_{LV} represents the surface tension of the droplet, R_b is the base radius of the droplet in contact with the solid. We assume that the water droplet on gradient surfaces can be considered as a spherical segment and the contact area is a circle with radius R_b, the initial volume of water droplet is V_o, thus the base radius can be calculated as:

$$R_b = \left(\frac{3V_0}{\pi}\frac{1}{2 - 3\cos\theta_d + \cos^3\theta_d}\right)^{1/3}\sin\theta_d \quad \text{(S5)}$$

As the wetting profile of gradient surface is discrete, the forces acting on the dynamic droplets were simplified and defined points at middle positions of two (four) adjacent wettability gradient regions were used to calculate the driving and hysteresis forces (Fig. S2).

The droplet just overlaps two wettability regions when moving towards hydrophobic regions (Fig. S2a). Therefore, the wettability gradient d$\cos\theta_d$ is the gradient between wettability regions S_{i+1} and S_{i+2} (the red and yellow contact line). And for the hydrophilic regions, droplet may overlap four wettability regions (Fig. S2b). The wettability gradient d$\cos\theta_d$ is divided into two parts: one is the gradient between S_{i+1} and S_{i+2} (the red and yellow contact line), and the other is the gradient between S_i and S_{i+3} (the blue contact line).

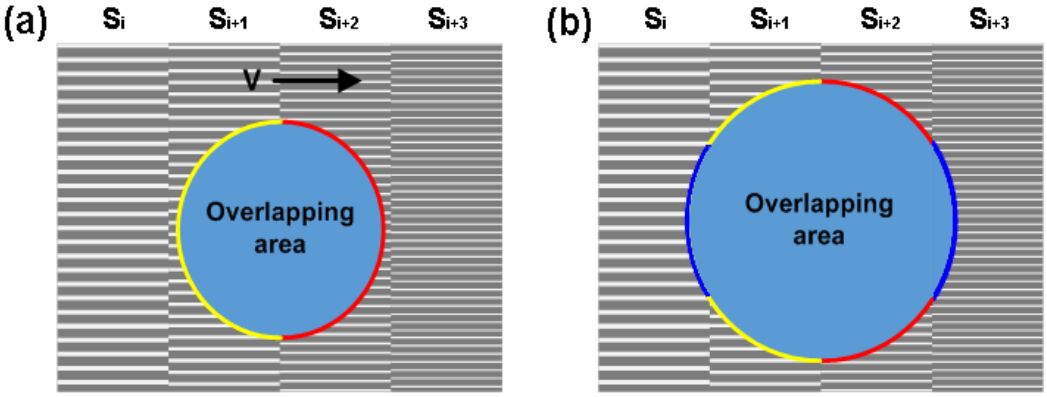

Figure S2. Defined droplet points at middle positions of two adjacent wettability gradient regions from S_i to S_{i+3}. (a) A droplet overlaps two wettability gradient regions for hydrophobic surface. (b) A droplet may overlap four wettability gradient regions for hydrophilic surface.

2. Viscous resistance force

The viscous resistance force can be calculated as $F_v = \int_A^B \sigma_{xz}(0) f r_w dx$, where $\sigma_{xz}(0)$ is the viscous stress at solid/liquid interface, f is the solid fraction wetted by water, r_w is the roughness for the flat region ($f\, r_w$ represents the total area being wetted). As from the surface without nano silicon pillars structure, $f = 1$.

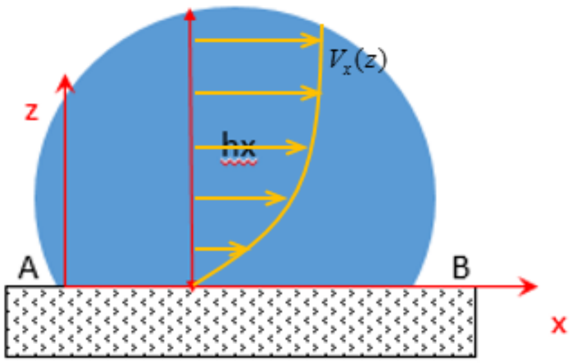

Figure S3. Schematic drawing shows a spherical segment water droplet ribbon with a circular across section.

From flow field analysis, we can get:

$$\sigma_{xz} = \eta \frac{\partial V_x(z)}{\partial z}|_{x=0} \tag{S6}$$

Where η is the viscosity of water, $V_x(z)$ shows the velocity as a function of z at position x (please refer to the yellow curved in Fig. S2). According to lubrication approximation

$$\frac{\partial P}{\partial x} = -\eta \frac{\partial^2 V_x(z)}{\partial^2 z} \tag{S7}$$

Where P is the pressure of Poiseuille flow, $\frac{\partial P}{\partial x}$ is the pressure gradient along x coordinate.

Combing with boundary condition: $V_x(0) = 0, \frac{\partial V_x}{\partial z}|_{z=h_x} = 0$. Thus,

$$Vh = \int_0^h V_x(z)dz \tag{S8}$$

$$V_x(z) = \frac{3V}{2h_x^2}(-z^2 + 2zh_x) \tag{S9}$$

Here, V is the average velocity of water droplet along z-axis, h_x is the height of droplet at point x (see Fig. S2). Thus,

$$\frac{\partial V_x(z)}{\partial z} = \frac{3V}{h_x^2}(-z + h_x) \tag{S10}$$

Finally,

$$\sigma_{xz} = \eta \frac{\partial V_x(z)}{\partial z}\Big|_{z=0} = \frac{3V\eta}{h_x} \tag{S11}$$

Thus, the drag force per unit length can be expressed as:

$$F_{vo} = 3\eta V \int_A^B \frac{f r_w dx}{h_x} \tag{S12}$$

If we assume that the flow field is uniform at solid/liquid interface, we can get the overall viscous resistance force:

$$F_v \approx \frac{F_{vo}}{2R}\pi R^2 = \frac{3}{2}\pi \mathrm{R}\eta V \int_A^{\mathrm{B}} \frac{f r_w dx}{h_x} \tag{S13}$$

where $f = \frac{L_1}{L}$, $r_w = \frac{L_1 + h}{L_1}$, L_1=90 μm, h=2 μm.

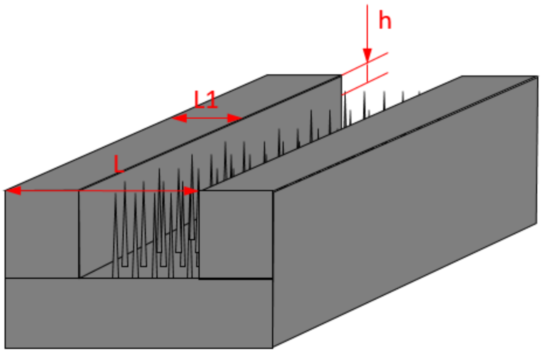

Figure S4. Schematic of the silicon nanopillars and SiO_2 plane

3. Pattern density and static CA

The droplet static contact angle on the composite pattern surface is provided by the Cassie-Baxter model.

$$\cos\theta_{\mathrm{CB}} = f\cos\theta_0 + f - 1 \tag{S14}$$

Here, θ_{CB} is the static CA of different pattern density surface and θ_0 is the intrinsic CA of SiO_2. The measured and calculated results as table S1 shows.

4. Droplet displacement and velocity

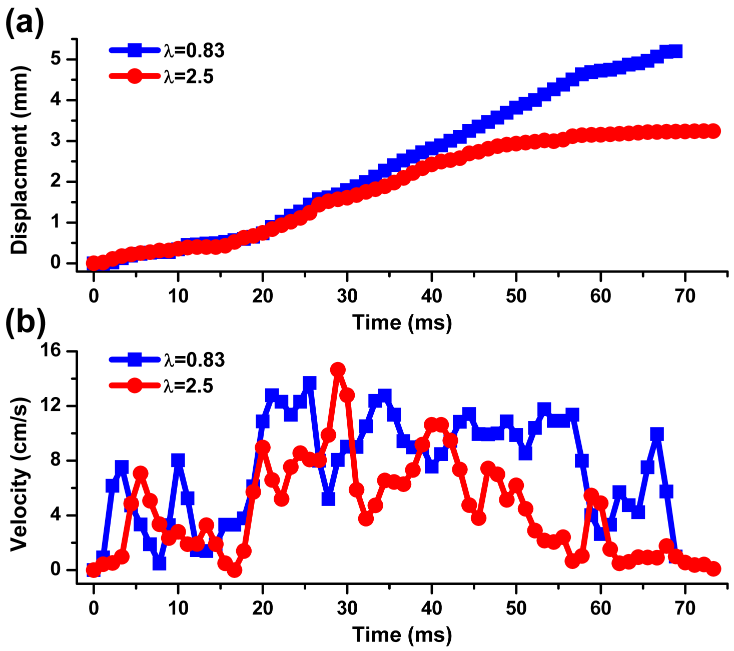

Figure S5. Droplet motion displacement (a) and velocity (b) on different wettability gradient surfaces.

5. Reproducibility of the wettability gradient surfaces.

We demonstrate the sample reproducibility on the radial path surfaces and the snapshots are shown as follow.

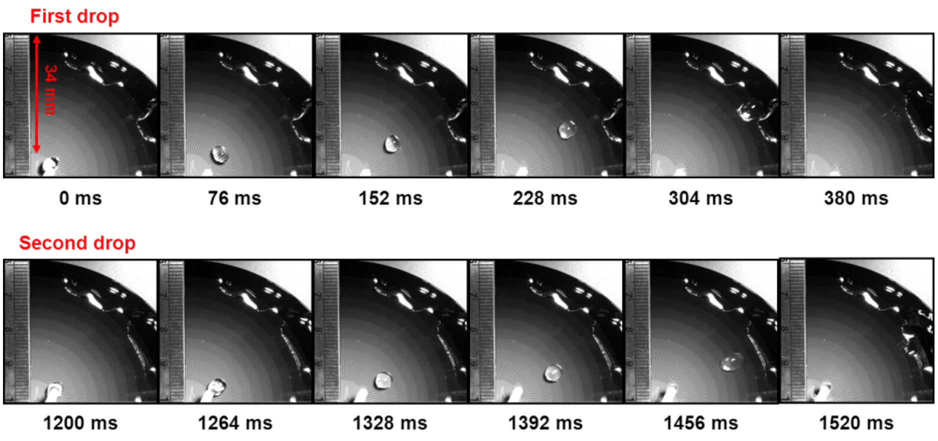

Figure S6. Droplet self-motion reproducibility of the wettability gradient surfaces.

Table S1. The measured and calculated Static CA.

**Pattern density (*f*)**	**Measurd Static CA (°)**	**Calculated Static CA (°)**
0.33	108.52	110.2078
0.40	101.88	102.3868
0.50	91.23	91.0393
0.59	81.93	81.0756
0.71	69.2	66.2554
0.91	39.4	38.2605
1	15.5	15.5000

Table S2.The reported wettability gradient range in different references.

	wettability gradient rangeθ(°)	Reference name	Journal	year
Ref. 1	About 15°<θ<95°	How to Make Water Run Uphill	Science	1992
Ref. 2	125°<θ<160°	Conversion of Surface Energy and Manipulation of a Single Droplet across Micropatterned Surfaces	Langmuir	2008
Ref. 10	30°<θ<110°	Molecular Combing of λ-DNA using Self-Propelled Water Droplets on Wettability Gradient Surfaces	ACS applied materials & interfaces	2016
Ref. 14	θ<100°	Automatic droplet transportation on a plastic microfluidic device having wettability gradient surface	Review of Scientific Instruments	2015
Ref. 15	25°<θ<110°	A microchip fabricated with a vapor-diffusion self-assembled-monolayer method to transport droplets across superhydrophobic to hydrophilic surfaces	Lab on a Chip	2010
Ref. 35	65.2°<θ<127.8°	Smart design of stripe-patterned gradient surfaces to control droplet motion	Langmuir	2011
Ref. 38	35°<θ<100°	Engineering functionality gradients by dip coating process in acceleration mode	ACS applied materials & interfaces	2014

Ref. 39	55°<θ<165°	Oil droplet self-transportation on oleophobic surfaces	Science Advances	2016
Ref. 40	θ<106°	Directional liquid spreading over chemically defined radial wettability gradients	ACS applied materials & interfaces	2012
Ref. 41	95°<θ<150°	Self-Propelling and Positioning of Droplets Using Continuous Topography Gradient Surface	Advanced Materials Interfaces	2014
Ref. 47	15°<θ<115°	Fabrication of Chemical Gradient Using Space Limited Plasma Oxidation and its Application for Droplet Motion	Advanced Functional Materials	2012
Ref. 50	10°<θ<80°	Rectified Motion of Liquid Drops on Gradient Surfaces Induced by Vibration	Langmuir	2002

Video legends

1. λ=0.83 wettability gradient surface.avi
2. λ=2.5 wettability gradient surface.avi
3. λ=0.83 wettability gradient surface without silicon nanopillar.avi
4. Annular pth.avi
5. Long straight path.avi
6. S path.avi
7. Droplet flow.avi

References

1. Daniel, S. & Chaudhury, M. K. Rectified motion of liquid drops on gradient surfaces induced by vibration. *Langmuir* 18, 3404-3407 (2002).
